# Estrogen, menopause, and Alzheimer’s disease: understanding the link to cognitive decline in women

**DOI:** 10.3389/fmolb.2025.1634302

**Published:** 2025-06-30

**Authors:** Nicholas Mervosh, Gayatri Devi

**Affiliations:** ^1^ Park Avenue Neurology, New York, NY, United States; ^2^ Departments of Neurology, Zucker School of Medicine, Northwell Health, New York, NY, United States; ^3^ Departments of Psychiatry, Zucker School of Medicine, Northwell Health, New York, NY, United States

**Keywords:** APOE genotype and dementia risk in women, precision medicine in women’s cognitive aging, hormone therapy and brain health, menopause related cognitive impairment, estrogen and Alzheimer’s disease

## Abstract

**Background:**

Women face a significantly higher lifetime risk of developing Alzheimer’s disease (AD) than men. This disparity is often attributed to longer female longevity, but growing evidence suggests a multifactorial origin, including hormonal, vascular, and immunologic contributions. Estrogen plays a critical neuroprotective role across multiple systems implicated in AD pathogenesis, including synaptic plasticity, mitochondrial function, and cerebrovascular integrity. However, clinical trials investigating hormone therapy (HT) for AD prevention have yielded mixed results, in part due to variability in study populations, timing of intervention, and formulation of hormones.

**Aims/methods:**

This review examines the biological rationale for estrogen’s role in cognitive aging, synthesizes clinical and translational data on hormone therapy and AD risk, and highlights the importance of vascular comorbidity, including cerebral small vessel disease, in mediating AD pathology.

**Conclusion:**

We propose that estrogen’s neuroprotective potential may be best realized in personalized treatment frameworks that account for age, timing, APOE genotype, and vascular burden. Interpretation of estrogen’s role in AD is further complicated by variability in diagnostic criteria, which may contribute to conflicting findings across studies. Recognition of menopause-related cognitive impairment as an early, hormonally modulated risk state may offer additional opportunity for timely intervention. Addressing this complexity is essential to refining AD prevention strategies in midlife women.

## 1 Introduction

Alzheimer’s disease (AD) disproportionately affects women, with nearly two-thirds of all diagnosed cases occurring in females. Biological, hormonal, and sociocultural factors are likely the primary drivers of the sex disparity in risk and clinical presentation, rather than increased longevity as previously believed (Beam et al., 2018; Jett et al., 2022; Vila-Castelar et al., 2023). Women not only exhibit a higher incidence and prevalence of AD but also tend to show faster cognitive decline and greater pathological burden once diagnosed (Lin et al., 2015).

Emerging research suggests that female-specific risk factors—especially those tied to hormonal transitions such as menopause—may critically influence brain aging and AD vulnerability (Toran-Allerand et al., 1999; Sherwin, 2009; Henderson and Sherwin, 2007). Estrogen, in particular, has garnered attention for its pleiotropic effects on neuronal resilience, cerebral perfusion, and immune modulation (Toran-Allerand et al., 1999). The perimenopausal and postmenopausal brain may thus experience a compound loss of estrogen’s protective influence at the very time when age-related neurodegeneration begins to accelerate (Henderson and Sherwin, 2007; Henderson and Rocca, 2012; Henderson et al., 2016). Interpretation of these findings is further complicated by inconsistencies in how AD is defined across the three major diagnostic frameworks, a factor that may obscure true associations between estrogen exposure and disease risk (Bieger et al., 2024).

Compounding this complex picture is the frequent co-occurrence of other primary brain co-pathology in aging women (Devi, 2023). AD almost never occurs in isolation. In fact, neuropathologic studies show that from 66% to 100% of AD cases exhibit coexisting primary brain pathologies—including vascular brain injury and Lewy body disease (Spina et al., 2021; Karanth et al., 2020). Mixed pathology is the norm, rather than the exception.

In addition to these structural and molecular risks, the menopausal transition itself is associated with measurable cognitive changes in a substantial proportion of women (Weber et al., 2014; Davis et al., 2015). Multiple longitudinal cohort studies demonstrate that up to 60% of midlife women report difficulties with memory, attention, and verbal fluency during perimenopause (Henderson and Sherwin, 2007; Weber et al., 2014; Davis et al., 2015). Objective testing confirms declines in verbal memory, working memory, and executive function, often correlating with fluctuations in estradiol and follicle-stimulating hormone (Davis et al., 2015). These changes, although subtle, can significantly impact quality of life and may be mistaken for early signs of dementia. Recognizing the menopausal origin of these symptoms is crucial for timely and appropriate management.

Menopause-related cognitive impairment (MeRCI), a recently defined clinical entity, describes the emergence of cognitive symptoms, including objective evidence of language, executive function and/or memory impairment, during the menopausal transition in the absence of other medical or psychiatric conditions (Devi, 2018; Maki and Henderson, 2012). MeRCI reflects the estrogen-sensitive cognitive phenotype often underrecognized in midlife women and may represent a modifiable early risk state within the Alzheimer’s disease continuum.

This review synthesizes current evidence on estrogen’s neuroprotective mechanisms, evaluates clinical trial data on hormone therapy and AD risk, and emphasizes the need to consider vascular comorbidity in both mechanistic understanding and therapeutic planning. By reframing AD as a spectrum disorder influenced by sex-specific biology and comorbid pathologies, we advocate for a precision medicine approach to prevention in midlife women.

## 2 Discussion

We begin by examining the multiple mechanisms through which estrogen may support neuronal health, including its influence on neuroplasticity, neurotransmitter regulation, oxidative stress, amyloid and tau pathology, neuroimmune function, and the integrity of the blood-brain barrier. Many of these areas have been extensively reviewed elsewhere and therefore will be summarized in this review.

We then discuss how the diagnostic criteria used in making a diagnosis of AD may significantly alter results of data. We review current evidence on estrogen’s role in cognitive impairment, drawing from both observational studies and randomized controlled trials. This includes an analysis of the specific estrogen formulations used, the cognitive effects of progestins, and the importance of the critical window for initiating hormone therapy.

Next, we explore the significant overlap between vascular and neurodegenerative pathology in women with Alzheimer’s disease and the potential modulatory role of estrogen in this context. We highlight the emerging clinical entity of menopause-related cognitive impairment, which may reflect a unique intersection of hormonal and neurodegenerative changes.

Finally, we discuss the clinical implications of these findings and consider how hormone therapy might be integrated into personalized treatment paradigms for women at risk of or experiencing cognitive decline.

### 2.1 Estrogen and the female brain: mechanisms of neuroprotection

Estrogen exerts extensive influence on the central nervous system (CNS), affecting not only reproductive regulation but also cognition, affect, and neuroprotection. Its effects are mediated through estrogen receptors—ERα and ERβ—widely distributed throughout the brain, including the hippocampus, prefrontal cortex, amygdala, and basal forebrain (McEwen et al., 2001; McEwen et al., 2012; Mitterling et al., 2010). These receptors facilitate both genomic transcriptional regulation and rapid non-genomic signaling, engaging intracellular cascades that impact brain aging and resilience to neurodegenerative stressors (McEwen, 2012; Ma et al., 2016).

#### 2.1.1 Synaptic plasticity, neurogenesis, and cognitive performance

Estrogen enhances synaptic connectivity through promotion of long-term potentiation (LTP), increased dendritic spine density, and heightened expression of synaptic proteins important for synaptic plasticity (Toran-Allerand et al., 1999; Henderson et al., 2016; Smejkalova and Woolley, 2010). These effects are especially pronounced in the hippocampus and prefrontal cortex—regions integral to working memory, spatial navigation, and executive function (Shanmugan and Epperson, 2014). Estradiol also stimulates adult neurogenesis in the dentate gyrus, a capacity that declines with age and estrogen deprivation (Barha and Galea, 2010; Grodstein et al., 2003). These synaptic and neurogenic effects underlie the cognitive advantages observed in premenopausal women and in animal models treated with estradiol (Mishra et al., 2023).

In mouse models, early ovarian failure during perimenopause enhances astrocyte activation and regional amyloid accumulation in the hippocampus during early-stage cerebral amyloid angiopathy, suggesting emerging neurovascular dysfunction, which is often correlated with the development of dementia (Platholi et al., 2023). Another mouse model study found that chemically induced perimenopause in mice with early-stage Alzheimer’s pathology increased amyloid-beta accumulation and heightened astrocyte and microglial activation in specific hippocampal subregions, although cognitive deficits were not yet apparent. These results support the idea that perimenopause represents a critical window of emerging vulnerability to Alzheimer’s-related brain changes where intervention may be beneficial (Marongiu et al., 2025).

#### 2.1.2 Neurotransmitter systems: acetylcholine, serotonin, dopamine, and norepinephrine

Estrogen exerts widespread influence on neurotransmitter systems critical to cognition, mood regulation, and behavioral flexibility—many of which are vulnerable to age-related decline and prominently implicated in AD. Estrogen receptors are expressed not only in cortical and hippocampal neurons but also in neurons producing acetylcholine and monoamines, including serotonin, dopamine, and norepinephrine (Ma et al., 2016; Ross and Van Bockstaele, 2021).

• Acetylcholine: Estrogen upregulates choline acetyltransferase, the enzyme responsible for acetylcholine synthesis, and enhances muscarinic receptor density in the basal forebrain and hippocampus. It also supports the integrity of cholinergic projections—among the earliest to deteriorate in AD—thereby preserving attention and memory processes. This cholinergic support underpins estrogen’s role in attention and memory (Russell et al., 2019; Newhouse and Dumas, 2015). The loss of this estrogenic support during perimenopause may compromise cholinergic tone and contribute to the early cognitive symptoms observed in prodromal AD (Kwakowsky et al., 2016).
• Serotonin: Estrogen modulates serotonergic function by increasing tryptophan hydroxylase expression—the rate-limiting enzyme in serotonin synthesis—and by regulating serotonin receptor subtypes, particularly 5-HT1A and 5-HT2A (MCEWEN Harold and Milliken, 2001; Amin et al., 2005; Bendis et al., 2024). These effects are associated with improvements in mood and affect and may explain the increased risk of depression during the menopausal transition (MCEWEN Harold and Milliken, 2001; Amin et al., 2005). Estrogen’s serotonergic actions are central to its role in maintaining emotional resilience and are increasingly recognized as part of its broader neuroprotective profile.
• Dopamine: Estrogen influences dopaminergic signaling by enhancing dopamine synthesis and turnover, and regulates D1 and D2 receptor expression, particularly in the prefrontal cortex, striatum, and nucleus accumbens. These regions govern executive function, working memory, motivation, and reward processing—domains commonly impaired in early AD (Almey et al., 2015). Membrane-associated estrogen receptors localized on dopaminergic neurons further modulate dopamine receptor expression and sensitivity (Bendis et al., 2024; Almey et al., 2015; Almey et al., 2012). These mechanisms underpin sex differences in dopamine-mediated cognition and highlight estrogen’s critical role in modulating frontostriatal function.
• Norepinephrine: Estrogen also modulates the locus coeruleus (LC) –norepinephrine system, which is involved in arousal, attention, vigilance, and stress regulation. Estrogen receptors are expressed in LC neurons, where estrogen preserves structural integrity and promotes norepinephrine synthesis and release. Notably, LC degeneration is one of the earliest pathologic changes in AD and contributes to neuroinflammatory priming and influences amyloid processing. Estrogen-mediated preservation of locus coeruleus function may be critical given its early degeneration in AD (Russell et al., 2019; Bangasser et al., 2016). Estrogen modulation of LC activity suggests another potential mechanism by which hormonal decline during perimenopause could amplify AD risk through disrupted arousal and neuroimmune regulation (Ross and Van Bockstaele, 2021).

Through this multifaceted support of key neurotransmitter systems, estrogen maintains the neurochemical infrastructure essential for cognitive and emotional regulation. The loss of estrogenic signaling during perimenopause may destabilize these systems, creating a period of heightened vulnerability to the neuropathological processes underlying Alzheimer’s disease.

#### 2.1.3 Mitochondrial function and oxidative stress

Mitochondria are central to neuronal metabolism, and their dysfunction is a key feature of aging and Alzheimer’s disease (McGill Percy et al., 2025). Estrogen enhances mitochondrial biogenesis, increases ATP production, and reduces oxidative stress through upregulation of antioxidant enzymes such as superoxide dismutase and glutathione peroxidase (Lejri et al., 2018; Velarde, 2013). This contributes to reduced accumulation of reactive oxygen species, which are implicated in both amyloid aggregation and tau hyperphosphorylation.

#### 2.1.4 Amyloid and tau pathophysiology

Estrogen influences amyloid precursor protein processing by promoting α-secretase activity and suppressing β-secretase, thereby favoring the non-amyloidogenic pathway (Cole and Vassar, 2007). *In vivo* models show that estrogen administration reduces Aβ levels and plaque formation and may reduce tau formation in post-menopausal women (Wang et al., 2024a; Henderson, 2014). Additionally, estrogen modulates kinases involved in tau phosphorylation, thereby mitigating neurofibrillary tangle development (Kantarci et al., 2016; Wang et al., 2024b; Depypere et al., 2023).

#### 2.1.5 Cerebrovascular and blood-brain barrier (BBB) integrity

Estrogen has vasoprotective effects, including the promotion of nitric oxide synthesis, improved endothelial function, and preservation of cerebral autoregulation (McNeill et al., 2002). It also maintains blood-brain barrier integrity, reducing permeability and neuroinflammatory infiltration (Maggioli et al., 2016). With estrogen loss, particularly during the menopausal transition, cerebral perfusion diminishes and the BBB becomes more vulnerable—conditions that may synergize with amyloid and vascular pathology to accelerate cognitive decline (Russell et al., 2019; Maggioli et al., 2016).

#### 2.1.6 Neuroimmune modulation

Microglia and astrocytes express estrogen receptors and are directly modulated by hormonal signaling (Mor et al., 1999). Estrogen promotes an anti-inflammatory glial phenotype, downregulates pro-inflammatory cytokines (e.g., IL-1β, IL-6, and TNF-α), and restrains chronic neuroinflammation—a hallmark of aging and AD (Mor et al., 1999; Villa et al., 2016). Estrogen deprivation in menopause may shift this balance toward a pro-inflammatory state, thereby exacerbating neuronal injury and synaptic loss (Villa et al., 2016).

### 2.2 Diagnostic criteria discordance and implications for research in HT and AD

Differences in Alzheimer’s disease diagnostic frameworks—particularly between the two biomarker-only National Institute on Aging–Alzheimer’s Association (NIA-AA) and the Alzheimer’s Association (AA) criterion, and the clinico-biological International Working Group (IWG) criteria—have important implications for estrogen research (Table 1) (Villain and Paretsky, 2024).

**TABLE 1:** Comparison of Alzheimer’s disease (AD) diagnostic frameworks.

Framework	Definition of AD	Are clinical symptoms required?	Biomarkers used
Alzheimer’s Association (2024 criteria)	AD is defined biologically based on abnormal Core biomarkers of AD	No	Core 1: Amyloid PET, CSF Aβ42, plasma p-tau217 Core 2: Tau PET, CSF p-tau
International Working Group criteria	AD is a clinical-biological syndrome requiring both a compatible phenotype and biomarker confirmation	Yes	CSF Aβ42, CSF total/p-tau, amyloid PET Supportive: hippocampal atrophy on MRI, FDG-PET
National Institute of Aging-Alzheimer’s Association ATN Framework (2011–2018)	AD defined by abnormal biomarkers: AT(N) with, or without symptoms	No for diagnosis Yes for clinical syndrome	A: Amyloid PET, CSF Aβ42 T: Tau PET, CSF p-tau N: FDG-PET, structural MRI, CSF total tau

CSF: cerebrospinal fluid. PET, positron emission tomography. FDG, fluorodeoxyglucose. ATN, amyloid tau neurodegeneration.

The IWG requires both clinical symptoms and biomarker evidence for diagnosis (Dubois et al., 2021). In contrast, the NIA-AA and the AA frameworks permit a biological diagnosis based solely on amyloid, tau, and neurodegeneration biomarkers (AT [N]), even in asymptomatic individuals (Brum et al., 2022; Jack et al., 2018). A third approach, endorsed by an Alzheimer’s Association-led working group, allows for diagnosis based on a single abnormal biomarker (Ja et al., 2024). When applied to the same cohort, these frameworks yielded a 42% discrepancy in diagnoses, especially in individuals with only one abnormal biomarker (Bieger et al., 2024). Notably, among individuals with isolated amyloid-beta abnormalities, 65% remained cognitively intact (Bieger et al., 2024; Villain and Paretsky, 2024).

For estrogen studies that rely on AD diagnosis as an outcome or grouping variable, such variability can distort associations, especially among cognitively normal, biomarker-positive midlife women. Researchers should therefore clearly specify which diagnostic criteria are used and consider those that integrate both clinical and biological features to improve relevance for sex-specific risk and treatment response.

### 2.3 The timing hypothesis and clinical trials of hormone therapy in Alzheimer’s disease

The “timing hypothesis” posits that the neuroprotective effects of estrogen are contingent upon when HT is initiated in relation to the onset of menopause. Specifically, estrogen may confer cognitive and structural brain benefits when administered during the critical window of perimenopause or early post-menopause, whereas delayed initiation—often a decade or more after menopause—may yield no benefit or even harm (Sherwin, 2009; Henderson and Rocca, 2012; Erickson et al., 2010; Lord et al., 2008).

#### 2.3.1 Observational evidence and the critical window

Early observational studies suggested a strong protective association between estrogen use and reduced AD risk. For example, data from the Cache County Study, which included persons aged 65 years or older, and the Baltimore Longitudinal Study on Aging, which included postmenopausal women ages 65 and older, indicated that women who used HT near the time of menopause had a lower incidence of AD (Shao et al., 2012; Nerattini et al., 2023). However, these studies were limited by healthy-user bias and lack of randomization.

The critical window hypothesis emerged from such findings, suggesting that the brain may retain estrogen sensitivity during a finite period following ovarian hormone withdrawal (Sherwin, 2009). Beyond this window, estrogen receptors may downregulate, and the brain may enter a pro-inflammatory, vulnerable state that does not respond favorably to exogenous hormones.

#### 2.3.2 Randomized controlled trials: mixed results

Randomized controlled trials have yielded mixed results and are briefly summarized in Table 2. The Women’s Health Initiative Memory Study (WHIMS) remains the most widely cited trial in the field Espeland et al. (2024). In this large, randomized controlled trial (RCT) of women aged 65 and older, conjugated equine estrogen (CEE) with or without medroxyprogesterone acetate (MPA) increased the risk of dementia and cognitive decline. However, this trial enrolled women well beyond the menopausal transition (mean age 69), raising questions about generalizability to midlife populations. Additionally, while there was an increased risk of all-cause dementia (including normal pressure hydrocephalus, for example,), there was NOT an increased risk for AD (Nerattini et al., 2023).

**TABLE 2:** Major clinical trials evaluating hormone therapy and cognitive outcomes in postmenopausal women.

Study	Population	Intervention	Key findings	Conclusion
WHIMS	Women ≥65 years	CEE ± MPA	Increased dementia risk	Late HT may be harmful
KEEPS-Cog	Women 42–58 years	Oral CEE or transdermal E2 + progesterone	No cognitive benefit	HT in early menopause may be neutral
ELITE	Women <6 vs. >10 years postmenopause	Oral estradiol ± vaginal progesterone	No cognitive benefit or harm	Supports timing hypothesis
WHIMSY	Women 50–55 years (WHI subset)	CEE vs placebo	No cognitive harm after 10 years	Early HT appears safe
KEEPS Follow-up	Post-KEEPS cohort ∼10 years later	CEE or E2 vs placebo	No long-term cognitive benefit or harm	HT may be neutral

CEE, conjugated equine estrogen. E2, estradiol. ELITE, early versus late intervention trial with estradiol. KEEPS-Cog, Kronos Early Estrogen Prevention Study -Cognitive Study. MPA, medroxyprogesterone acetate. WHI, women’s health initiative. WHIMS, Women’s Health Initiative Memory Study. WHIMSY, Women’s Health Initiative Memory Study of Younger Women.

Subsequent RCTs have painted a more nuanced picture:

• The KEEPS-Cog trial studied younger postmenopausal women (ages 42–58) and found no adverse cognitive effects of transdermal estradiol over 4 years, although it was not powered to detect AD incidence (Gleason et al., 2015).
• The ELITE study stratified women by time since menopause and found that estradiol improved carotid artery intima-media thickness in recently menopausal women (<6 years) but not in those further out (>10 years), indirectly supporting the timing hypothesis for vascular aging (Hodis et al., 2016).
• The WHIMSY trial, a follow-up to WHI, evaluated cognition in women who had received CEE between ages 50 and 55. It found no increased risk of cognitive impairment over 10 years of follow-up, suggesting that early use of estrogen may be neutral or beneficial Espeland et al. (2024); Maki and Henderson, 2012; Shumaker et al., 2004).

#### 2.3.3 Formulation, route, and progestin effects

Not all estrogens or regimens are equivalent. The WHIMS trial employed oral CEE, a formulation derived from pregnant mare’s urine, along with MPA, a synthetic progestin Espeland et al. (2024); Shumaker et al., 2004). In contrast, bioidentical 17β-estradiol—particularly via transdermal routes—may have more favorable effects on cognition and cardiovascular health. Oral estrogens undergo first-pass hepatic metabolism, increasing coagulation factors and inflammatory markers, whereas transdermal estradiol bypasses the liver and maintains a more physiologic estrogen profile (Kantarci et al., 2016; Wharton et al., 2011).

Progestin co-administration further complicates interpretation. While necessary for endometrial protection in women with a uterus, synthetic progestins like MPA may attenuate estrogen’s beneficial effects (Stanczyk et al., 2013). Natural micronized progesterone appears to have a more favorable safety and neurocognitive profile but has not been extensively studied in long-term AD prevention trials.

These differences were directly explored in the Kronos Early Estrogen Prevention Study (KEEPS), which randomized healthy recently postmenopausal women (median age 42–58 years old; within 3 years of menopause) to either oral CEE, transdermal 17β-estradiol, or placebo, all with cyclic micronized progesterone (Gleason et al., 2015; Gleason et al., 2024). The KEEPS-Cog sub-study evaluated cognitive outcomes over 4 years and found no cognitive benefit from either formulation compared to placebo. Although neither route caused harm, the anticipated cognitive enhancement—particularly from transdermal estradiol—was not observed, challenging assumptions that bioidentical transdermal therapy would outperform CEE in preserving cognitive function (Gleason et al., 2024).

A long-term follow-up study of the KEEPS cohort approximately 10 years after cessation of therapy similarly found no lasting benefit or harm on cognitive outcomes from either hormone regimen. These findings suggest that in healthy, low-risk women, even early HT initiation may be cognitively neutral, and that timing, formulation, and population risk profile all influence therapeutic outcomes (Gleason et al., 2024).

This nuanced evidence underscores the importance of tailoring hormone therapy—not only in terms of age and timing, but also formulation, route, and individual risk factors, including cardiovascular and genetic vulnerability.

#### 2.3.4 Limitations and remaining questions

Despite promising mechanistic data, current clinical trials remain underpowered, short in duration, and heterogeneous in design. Most have not targeted women at the highest risk—those with metabolic syndrome, cerebrovascular pathology, or APOE-ε4 genotype (Saleh et al., 2023). Furthermore, outcomes have focused on global cognition rather than sensitive biomarkers such as amyloid PET imaging, tau load, white matter hyperintensity volume, or regional perfusion.

Another prospective cohort of women tracked from midlife to late life, early initiation of hormone therapy—particularly within 5 years of menopause—was associated with reduced risk of cognitive impairment and dementia. Notably, the protective association was strongest in APOE-ε4 non-carriers, whereas APOE-ε4 carriers did not appear to benefit and, in some cases, showed potential harm with later or prolonged hormone use (Taxier et al., 2022a).

This study reinforces both the critical window hypothesis and the need for genotype-informed HT decision-making, aligning with other studies showing that APOE-ε4 carriers may have heightened sensitivity to hormonal and metabolic stressors. These findings argue for tailoring both timing and duration of hormone therapy to genetic and vascular risk (Saleh et al., 2023; Taxier et al., 2022a).

Such observational data, while subject to confounding factors, offer valuable direction for future trials and underscore the need for stratified prevention strategies.

### 2.4 The overlap of vascular and neurodegenerative pathology in women with Alzheimer’s disease

Alzheimer’s disease is almost never a pure neuropathological entity (Devi, 2023). Recent studies, including large autopsy series and biomarker-based analyses, confirm that nearly all cases of AD have coexisting primary brain pathology. Common vascular comorbities include cerebral small vessel disease (SVD), microinfarcts, white matter hyperintensities (WMHs), and arteriolosclerosis (Hodis et al., 2016; Simpkins et al., 2009). Vascular comorbidities are particularly prevalent in women, amplifying cognitive impairment and complicating diagnostic and therapeutic strategies.

#### 2.4.1 Vascular aging and estrogen loss

Estrogen plays a central role in maintaining cerebrovascular health through its effects on endothelial function, nitric oxide production, arterial compliance, and blood-brain barrier (BBB) integrity (Maggioli et al., 2016). With the menopausal transition, the decline in estrogen leads to increased arterial stiffness, endothelial dysfunction, and enhanced vulnerability to ischemic injury. These vascular changes may precede or exacerbate neurodegenerative processes, particularly in women with underlying metabolic or hypertensive risk (Hodis et al., 2016).

Women also appear to have greater WMH burden than men for a given vascular risk profile, and WMHs are a well-established predictor of cognitive decline, especially executive dysfunction and processing speed—domains often affected early in women with AD (Hodis et al., 2016).

#### 2.4.2 Interaction between vascular pathology and amyloid-tau cascade

Vascular dysfunction may synergize with amyloid and tau pathology to accelerate neurodegeneration. Chronic hypoperfusion impairs glymphatic clearance of Aβ, while blood-brain barrier breakdown facilitates neuroinflammation and toxic protein accumulation. Additionally, microvascular disease may sensitize neurons to injury from tau hyperphosphorylation.

In women, this interplay may be intensified by estrogen loss. For example, animal models of ovariectomy show increased cerebrovascular inflammation, reduced perfusion, and enhanced Aβ deposition—effects partially reversed by estrogen replacement (Hodis et al., 2016; Simpkins et al., 2009; Attems et al., 2014). These models suggest that estrogen’s protective role includes dampening the vascular contributions to AD pathology.

#### 2.4.3 Cerebrovascular burden as a modifier of hormone therapy response

Vascular burden may also modulate the cognitive response to hormone therapy. Subgroup analyses from the WHIMS-MRI study revealed that older women on hormone therapy had increased WMH volume, particularly those with preexisting vascular risk factors (Coker et al., 2009). This has raised concern that in the presence of significant SVD, exogenous estrogen may exacerbate white matter injury, particularly when initiated late.

Conversely, studies in younger women with lower vascular burden suggest more neutral or even beneficial effects on brain perfusion and connectivity. These findings support a precision-medicine approach in which vascular status—assessed via imaging or biomarkers—guides HT candidacy and regimen.

#### 2.4.4 Racial and metabolic disparities

It is important to recognize that vascular risk and estrogen deficiency intersect with social determinants of health, leading to disproportionate burden in Black and Hispanic women, who have higher rates of hypertension, diabetes, and stroke (Chinn et al., 2020). These populations are underrepresented in clinical trials, yet their risk profiles may make them more susceptible to both the benefits and harms of HT.

Incorporating vascular screening and risk stratification—particularly in midlife—may identify women most likely to benefit from early estrogen intervention and those in whom non-hormonal vascular risk management should take precedence.

### 2.5 Menopause-related cognitive impairment (MeRCI): a distinct clinical entity

Menopause-related cognitive impairment (MeRCI) is a recognized syndrome describing cognitive deficits that emerge during the perimenopausal and early postmenopausal transition, often in otherwise healthy women. Symptoms commonly include reduced verbal fluency, memory lapses, and executive dysfunction, often occurring in the absence of mood disorders or clear structural brain abnormalities (Devi, 2018).

Studies show that 34%–62% of midlife women report memory changes during menopause (Devi et al., 2005; Drogos et al., 2013). These subjective complaints correlate with objective reductions in verbal memory and fluency on neuropsychological testing (Sherwin, 2009; Henderson and Sherwin, 2007). Critically, these deficits can mimic early signs of neurodegenerative disease—leading to misdiagnoses such as Alzheimer’s disease or frontotemporal dementia (Henderson and Sherwin, 2007; Weber et al., 2014; Devi, 2018; Leblanc et al., 2002).

Diagnostic criteria for MeRCI include subjective and objective cognitive changes, as noted in Table 3, temporal association with menstrual irregularity, and exclusion of other medical or psychiatric causes (Devi, 2018). Brain imaging is often unremarkable, and symptoms may stabilize or improve with short-term hormone therapy or cognitive remediation strategies (Devi, 2018).

**TABLE 3:** Diagnostic criteria for menopause-related cognitive impairment (MeRCI).

• Subjective change in cognition reported by the patient • Occurs during the menopausal transition, defined by persistent change in frequency and quality of menses for ≥12 months • Not attributable to other medical conditions, medications, psychiatric illness, or dementia • Objective evidence of decline in one or more cognitive domains (e.g., memory, verbal fluency, executive function), beyond age-expected norms • Laboratory evidence of perimenopause (e.g., elevated Follicle Stimulating Hormone) may support the diagnosis but is not required

Because MeRCI is underrecognized in routine neurological and gynecological evaluations, many affected women receive incorrect diagnoses or unnecessary treatment. Routine screening for cognitive changes during menopause should be incorporated into clinical practice, particularly in women reporting difficulty with language, memory, or multitasking. Early recognition can prevent stigmatizing misdiagnoses and facilitate appropriate interventions.

### 2.6 Integrating hormone therapy into Alzheimer’s prevention: future directions and clinical considerations

While the neuroprotective potential of estrogen is well-supported by preclinical data, translation into effective clinical prevention of Alzheimer’s disease has proven complex. Disparate trial results reflect the challenges of a “one-size-fits-all” approach to hormone therapy and underscore the need for precision targeting based on age, timing, genotype, vascular health, and formulation.

#### 2.6.1 The need for risk-stratified prevention frameworks

Future prevention strategies should be individualized, not only by menopausal timing but also by risk phenotype. For example, a recently postmenopausal woman with a strong family history of AD, APOE-ε4 positivity, and minimal vascular burden may derive benefit from early transdermal estradiol, particularly if initiated within 5 years of menopause (Kantarci et al., 2016). In contrast, an older woman with metabolic syndrome, hypertension, and white matter hyperintensities may be better served by aggressive vascular risk reduction rather than hormone therapy.

This approach requires integrating biomarkers into decision-making, such as:

• Neuroimaging markers: hippocampal atrophy, cerebral perfusion, PET tau and amyloid (Coughlan et al., 2022; Coughlan et al., 2023; Coughlan et al., 2025).
• Plasma or CSF biomarkers: Aβ42/40, phosphorylated tau, neurofilament light (Coughlan et al., 2025).
• Genetic markers: Apolipoprotein E genotype and other AD risk polymorphisms (Saleh et al., 2023; Wang and Brinton, 2016; Taxier et al., 2022b).

#### 2.6.2 Opportunities for combination and sequential therapies

Estrogen’s failure to show consistent benefit in isolation may reflect a need for combination strategies. Potential synergistic approaches include:

• HT + lifestyle intervention: Exercise, Mediterranean diet, and cognitive training may amplify HT effects on brain plasticity.
• HT + anti-hypertensive or anti-diabetic agents: Addressing vascular risk may unmask the cognitive benefits of HT.
• Sequential or cycling therapy: Intermittent HT may retain efficacy while minimizing long-term exposure risks.

Such strategies should be tested in next-generation trials that use brain biomarkers as primary outcomes and recruit enriched populations at midlife.

#### 2.6.3 Clinical counseling: communicating nuance

For clinicians, translating this nuanced landscape into practice requires transparent, individualized risk-benefit counseling. Core principles include:

• Timing matters: Benefits are more likely when HT is initiated close to menopause onset (Hodis et al., 2016).
• Formulation matters: Transdermal estradiol and micronized progesterone are preferable to oral CEE and synthetic progestins (MŠ et al., 2023).
• Personal risk profile matters: APOE-ε4 status, vascular comorbidities, and cognitive symptoms must inform decisions (Riedel et al., 2016).

Hormone therapy should not be universally recommended for AD prevention, but neither should it be categorically dismissed—particularly in healthy women at midlife (Wharton et al., 2011). Reframing the conversation from binary risk to contextual, individualized potential empowers women to make informed choices.

#### 2.6.4 Research priorities

Key directions for future investigation include:

• Large, long-duration trials of transdermal estradiol in at-risk midlife women, stratified by APOE status and vascular health
• Integration of neuroimaging and fluid biomarkers into HT studies
• Inclusion of diverse populations, especially underrepresented racial and ethnic groups
• Evaluation of brain outcomes beyond global cognition, such as hippocampal volume, WMHs, network connectivity, and centiloid clearance

Only through such refined approaches can we resolve the longstanding ambiguity around estrogen and AD and deliver truly personalized prevention to the women most at risk.

## 3 Conclusion

Alzheimer’s disease in women is not simply a consequence of living longer—it is a reflection of complex, sex-specific biological, vascular, and hormonal interactions that begin decades before clinical onset. The abrupt decline in estrogen during menopause appears to be a pivotal inflection point in this trajectory, contributing to measurable cognitive changes, modulation of neurotransmitter systems, vascular vulnerability, and the acceleration of AD-related pathology.

Yet, estrogen’s therapeutic potential has been obscured by inconsistencies in study design, population selection, and timing of intervention. Evidence suggests that hormone therapy may offer cognitive benefits—particularly when initiated early in the menopausal transition, using bioidentical formulations and appropriate delivery routes. Conversely, delayed or inappropriate hormone use may fail to protect or may even harm, especially in the context of existing vascular disease or APOE-ε4 carrier status.

Future studies should clearly define the diagnostic criteria used for AD, as differences in classification frameworks may significantly affect both participant selection and interpretation of estrogen-related outcomes.

Recognition of menopause-related cognitive impairment as a distinct clinical entity offers a valuable framework for identifying estrogen-sensitive cognitive decline and avoiding misdiagnosis. Integrating vascular risk profiling, neuroimaging, genotype information, and menopausal timing into cognitive assessments will allow clinicians to tailor better interventions.

Hormone therapy should not be universally recommended for Alzheimer’s prevention, but neither should it be uniformly dismissed. A nuanced, precision medicine approach—targeting the right women, at the right time, with the right formulation—holds promise for altering the course of cognitive aging in women. The next-generation of trials must rise to this complexity, and clinical practice must evolve to reflect it.
